# Knowledge, attitudes, and practices regarding antibiotic use and resistance among health science and non-health science university students in Thailand

**DOI:** 10.1371/journal.pone.0296822

**Published:** 2024-01-05

**Authors:** Nopadol Precha, Suppachai Sukmai, Muhammadsarif Hengbaru, Muhammadkaddfee Chekoh, Sawanya Laohaprapanon, Prasert Makkaew, Nazri Che Dom

**Affiliations:** 1 Department of Environmental Health and Technology, School of Public Health, Walailak University, Nakhon Si Thammarat, Thailand; 2 Excellent Center for Dengue and Community Public Health, Walailak University, Nakhon Si Thammarat, Thailand; 3 Centre of Environmental Health and Safety Studies, Facultyof Health Sciences, Universiti Teknologi MARA (UiTM), Selangor, Malaysia; 4 Integrated Mosquito Research Group (I-MeRGe), Universiti Teknologi MARA (UiTM), Selangor, Malaysia; 5 Institute for Biodiversity and Sustainable Development (IBSD), Universiti Teknologi MARA (UiTM), Selangor, Malaysia; University of Ilorin, NIGERIA

## Abstract

Antibiotic-resistant bacteria (ARB) have been recognized as one of the global health issues affecting humans, animals, and the environment. A lack of knowledge, negative attitudes, and irrational drug use can make significant contributions to the spread of ARB. This study aimed to assess the knowledge, attitudes, and practices (KAP) regarding antibiotic use and resistance among health science (HS) and non-health science (NHS) students and to determine the factors that influence their KAP concerning antibiotic use and resistance. A cross-sectional study was conducted among 404 HS and NHS students in Southern Thailand from December 2021 to March 2022. The students who fulfilled the study inclusion criteria responded to a questionnaire that had five dimensions. Descriptive statistics were used to analyze the qualitative variables, and Fisher’s exact test was applied to compare the demographic variables, KAP responses between the HS and NHS students. The KAP regarding antibiotic use and resistance for each variable were compared using the Mann–Whitney *U* test and Kruskal–Wallis *H* test. Spearman’s correlation test was used to estimate the correlation between the variables and KAP. A total of 404 (HS,162; NHS,242) students completed the self-administered questionnaire. The students’ highest score was for attitude, followed by practice and knowledge. Our findings revealed that the HS students had higher levels of KAP correlated with antibiotic use and resistance than the NHS students (P < 0.001). The higher KAP scores were among the more senior students, which indicates that instruction on antibiotics was effective in their curriculum. Antibiotic use and resistance knowledge and attitudes should be conveyed to all university students via academic curriculum. Such interventions could set the standard for rational antibiotic use as well as long-term prevention and control of antibiotic-resistant bacteria.

## Introduction

Over the last decades, antibiotic resistant bacteria (ARB) has emerged as one of the global health issues, which widely affects in human, animals and environments [1, 2]. The rise of antibiotic resistance, which can limit the effectiveness of infectious diseases treatment, is one of the consequences of inappropriate antibiotic use. Inadequate dosage, use of antibiotics for non-bacterial infections, inappropriate self-medication, and nonadherence to dosing regimens have been recognized as significant global contributors to antibiotic-resistant bacteria (ARB) in veterinary, human, and agricultural settings [3–7]. Furthermore, ARB is growing and spreading as a result of self-medication, incorrect prescription, inappropriate consumption, and overuse of antimicrobial drugs. Antibiotics are frequently used inappropriately in the treatment of non-infectious and infectious diseases caused by pathogens other than bacteria, and self-medication is common in developing countries [4, 8, 9]. In Thailand, legislation allows community pharmacists to dispense antibiotics without a prescription, enabling people to purchase antibiotics without a doctor’s input [10]. In Thailand, the regulations regarding the availability of antibiotics without a prescription may differ from those in some other countries. Pharmacists are authorized to dispense antibiotics after a brief consultation under their professional judgment. A lack of public knowledge about antibiotic use, attitudes toward antibiotic use and resistance, easy access to antibiotics in many places, and a lack of awareness of the policies regarding rational antibiotic use are all factors that can influence irrational antibiotic use [11, 12].

Antibiotic knowledge, attitudes, and practices were examined across various population sectors, encompassing community members, pharmacists, nurses, and university students [13–22]. Notably, healthcare students have been identified as a pivotal population capable of making substantial contributions in transferring comprehensive antibiotic knowledge, fostering appropriate attitudes, and practicing responsible antibiotic use to enhance patient care and improve healthcare outcomes [19, 23, 24]. A study of public health students from 18 Chinese universities revealed a positive association between attitudes and knowledge and knowledge and practices regarding antibiotic use and resistance [25]. Studies conducted in various parts of the world have revealed that inappropriate antibiotic use among university students is caused by self-medication and a lack of knowledge about antibiotic use [7, 15, 17, 19, 25–31]. Furthermore, a comparative study found that the levels of KAP pertaining to antibiotic use and resistance were significantly higher among health-related major students than non-health-related major students [17, 30, 32–34]. An assessment of the KAP pertaining to antibiotic use and resistance among university students could serve as a valuable tool in identifying gaps or barriers to behavior change, which in turn can inform the development of targeted interventions aimed at preventing the spread of antibiotic-resistant bacteria (ARB) and promoting rational antibiotic use [34].

According to Thailand’s national household survey of antibiotic knowledge and use conducted in 2017 and 2019, there are significant gaps in the public’s knowledge and practices in relation to antibiotic use [14, 18]. One of the recommendations from these surveys was that specific information on the rational use of antibiotics and antimicrobial resistance (AMR) should be promoted to groups other than those with low education. As previously described, students are recognized as key contributors to antimicrobial stewardship through their engagement in educational initiatives, adherence to guidelines, effective communication with patients and healthcare professionals, participation in surveillance and monitoring activities and collaboration with multidisciplinary teams. Health science (HS) students, in particular, are the future health professionals who will be responsible for antibiotic use and resistance control. Although non-health science (NHS) students may not take direct action on antibiotic issues in their work, they will also be among antibiotic users.

The goals of Thailand’s National Strategic Plan on Antimicrobial Resistance 2017–2021, which is one of the country’s most significant strategic plans to combat antibiotic resistance, are to increase public understanding of antibiotics and raise awareness of rational antibiotic use [35–37]. This study aimed to evaluate the KAP of HS and NHS students in relation to antibiotic use and resistance, as well as to identify the factors that influence their KAP regarding antibiotic use and resistance. The findings of this study will provide valuable insights for developing strategies to enhance the KAP concerning antibiotic use and resistance in the specific target population. Furthermore, these findings contribute to the advancement of antimicrobial stewardship, which encompasses a comprehensive approach aimed at optimizing antimicrobial use, improving patient care outcomes, and reducing the emergence of antimicrobial resistance.

## Materials and methods

### Study design

A cross-sectional design was used to investigate the KAP regarding antibiotic use and resistance among HS and NHS university students in Southern Thailand. The KAP information survey was carried out from December 2021 to March 2022.

### Ethical approval

The study protocol was approved by the Human Research Ethics Committee of Walailak University (WU; Reference number: WUEC-21-343-01). Written informed consent was obtained from participants before participation in the study. The objectives and other important information regarding the study were explained to them. Confidentiality was strictly upheld throughout the research process. To maintain confidentiality, measures such as anonymization of questionnaires and de-identification of participant information were implemented. Addition, this study was performed in accordance with relevant guidelines and regulations.

### Study population

Walailak University (WU), located in the southern region of Thailand, is a public university comprising 17 schools/colleges and one college of graduate studies. During the study period, the university had 8,652 undergraduate students. The institution offers diverse programs encompassing health science, science and technology, and social sciences. Notably, it is essential to acknowledge that the duration of study programs varies, with the School of Medicine and Pharmacy following a 6-year curriculum. Additionally, the two colleges (Akkhraratchakumari Veterinary College and International College of Dentistry) had only first to third-year students as they were recently established. To minimize bias from clinical-level student experiences, this study specifically recruited participants from the first to fourth years of study.

### Sample size

A sample size of 367 subjects was calculated using the Krejcie and Morgan formula:

$$n=\frac{x^{2}Np(1-p)}{e^{2\left(N-1\right)\;+}x^{2}p\;(1-p)}$$

The sample size was based on a reference population of 8,652 students in the university at a 95% confidence level, a 5% margin of error, and response distribution of 0.5. We anticipated dropout data, so sampling corrections of 5% were included in the sample size calculation. Consequently, a total of 404 students were recruited for the study, including 242 NHS students and 162 HS students. These numbers reflect a proportion of approximately 1:1.5 between the HS and NHS students.

### Sampling method

A total of 8,652 undergraduate students from all the schools at WU were recruited using purposive sampling. The students needed to be at least 18 years old, enrolled as a first- to fourth-year undergraduate student, and willing to participate after reading the study’s informed consent statement. The students who completed the online survey but indicated that they were not enrolled undergraduates, were under the age of 18, did not sign the informed consent form, or provided incomplete response data were excluded from the analysis.

### Questionnaire design

A self-administered questionnaire was designed after an evaluation of previous studies regarding the KAP pertaining to antibiotic use and resistance, and it was subsequently modified to cover all the important points of this study [15, 20, 22]. The questionnaire was reviewed and evaluated by three experts in the fields of pharmacy, medical technology, and public health to validate its content, relevance, and readability. The questionnaire was revised in response to the experts’ advice. The questionnaire was modified for content validity until a content validity index of 0.7 or higher was obtained. The content validity index value of the questionnaire used in this study was 0.82, which indicated that the content validity of the survey was acceptable [38, 39]. A pilot study of 30 HS and NHS students was conducted to improve the final version of the questionnaire. The students who participated in the pilot study were not included in the final analysis. Cronbach’s alpha was used to calculate the questionnaire’s reliability, and the questionnaire had an acceptable Cronbach’s alpha of 0.8.

The questionnaire was written in Thai and contained 51 questions divided into five sections. The first section consisted of five questions that covered the respondents’ demographic information, such as gender, age, religion, school, and study year. The second section focused on their history of illness and antibiotic consumption and procurement in the previous 12 months as well as their sources of antibiotic use and resistance knowledge. The third section, which included 18 questions, assessed the respondents’ knowledge of antibiotic use and resistance. Categorical responses of “yes” or “no” were used in the questions that evaluated the respondents’ knowledge of antibiotic use and resistance. Each correct answer was allocated one point, and each incorrect answer was assigned zero points. The fourth section addressed attitudes toward antibiotic use and resistance. It included 10 statements comprising both correct and incorrect statements. The answers were graded on a five-point scale for the correct statements: largely agree = 5, agree = 4, uncertain = 3, disagree = 2, and largely disagree = 1. For the incorrect statements, the answers were reverse graded. In the fifth section of the questionnaire, 12 questions were used to evaluate antibiotic self-practice, health education, and antibiotic residual management. The questions regarding appropriate practices were graded with 0 points for “never,” 1 for “sometimes,” and 2 for “always,” while the questions about inappropriate practices were reverse graded.

### Data collection

The data were collected using an online questionnaire platform. All the relevant information regarding the study, including the title, objectives, informed consent form, and KAP questions, was designed and generated via Google Forms. A link to the self-administered questionnaire was distributed to each student via the university email platform and Facebook. The survey was available online for one month (i.e., December 20, 2021–January 20, 2022). After receiving the completed questionnaires from 162 HS and 242 NHS students, the data acquisition system was closed. The questionnaires with incomplete responses were deemed invalid.

### Data analysis

The completed questionnaire data were verified and then analyzed using IBM SPSS Statistics. The descriptive statistics and Fisher’s exact test were used to analyze the qualitative variables and compare the characteristics of the HS and NHS students, while the quantitative variables were summarized using mean ± standard deviation (SD). In terms of interpreting the results of the KAP questions using the scoring scheme, a score that was higher than 80% was considered acceptable, a score between 60% and 80% was considered moderate, and a score less than 60% was considered low [29]. Spearman’s correlation test was used to estimate the relationships between the students’ KAP. The indexes of KAP were transformed to a scale ranging from 0 (worst possible score) to 100 (best possible score) as follows [40]:

$$\begin{array}{l} \text{Total}\;\text{score}=([\text{score}\;\text{obtained}-\text{lowest}\;\text{possible}\;\text{score})/\\ (\text{maximum}\;\text{possible}\;\text{score}-\text{minimum}\;\text{possible}\;\text{score}])\times 100. \end{array}$$

The Mann–Whitney *U* and Kruskal–Wallis *H* tests were used to compare the KAP indexes based on the students’ sociodemographic features (study program, gender, academic year, religion), antibiotic consumption (antibiotic consumption in the past year), and education received (training or workshops). A P-value less than 0.05 was considered statistically significant.

## Results

### Socio-demographic characteristics

We investigated the KAP related to antibiotic use and resistance among HS and NHS students from December 2021 to March 2022. A total of 404 participants completed the questionnaire and were divided into two groups based on their major programs: 40.10% (n = 162) were majoring in one of the following health science programs: Medicine, Pharmacy, Nursing, Allied Health Science, and Public Health. The remaining 59.90% of the students (n = 242) were studying NHS programs (Art, Science, Political Science and Law, Management, Informatics, Agricultural Technology and Food, Engineering and Technology and Architecture and Design) (Table 1). The analysis revealed significant differences in gender, religion, age, and academic year between HS and NHS students. However, no statistically significant differences were observed regarding training or workshop on antibiotic use between on antibiotic use between the HS and NHS students. In terms of overall health, it was found that 58.66% (237/404) of the students had been ill in the previous 12 months. The most common health issues reported were flu (48.27%, 195/404), followed by diarrhea (18.31%, 74/404), tonsillitis (8.41%, 34/404), and gastritis (5.45%, 22/404).

**Table 1:** Demographic characteristics of participants.

Characteristics	% (n) Total (N = 404)	% (n) HS (N = 162)	% (n) NHS (N = 242)	*P* (Fisher’s Test)
**Gender**				
Male	39.60% (160)	30.86% (50)	45.45% (110)	0.004
Female	60.40% (244)	69.14% (112)	54.55% (132)	
**Religion**				
Buddhism	78.47% (317)	74.69% (121)	80.99% (196)	0.014
Islam	20.05% (81)	25.31% (41)	16.53% (40)	
Other	1.48% (6)	0.00% (0)	2.48% (6)	
**Age (Mean±S.D.)**	20.76 ± 1.20	20.64 ± 1.29	20.85 ± 1.14	
18–19	16.83% (68)	22.84% (37)	12.81% (31)	0.009
20–21	52.48% (212)	44.44% (72)	57.85% (140)	
> 22	30.69% (124)	32.72% (53)	29.34% (71)	
**Academic year**				
1	12.87% (52)	18.52% (30)	9.09% (22)	<0.001
2	18.56% (75)	17.90% (29)	19.01% (46)	
3	28.22% (114)	14.20% (23)	37.60% (91)	
4	40.35% (163)	49.38% (80)	34.30% (83)	
**School**				
Art	5.94% (24)	-	9.92% (24)	
Science	1.73% (7)	-	2.89% (7)	
Political Science and Law	16.58% (67)	-	27.69% (67)	
Management	8.66% (35)	-	14.46% (35)	
Informatics	5.45% (22)	-	9.09% (22)	
Agricultural Technology and Food	2.48% (10)	-	4.13% (10)	
Engineering and Technology	14.36% (58)	-	23.97% (58)	
Architecture and Design	4.70% (19)	-	7.85% (19)	
Nursing	3.96% (16)	9.88% (16)	-	
Pharmacy	4.46% (18)	11.11% (18)	-	
Medicine	1.49% (6)	3.70% (6)	-	
Allied Health Science	8.17% (33)	20.37% (33)	-	
Public Health	22.03% (89)	54.94% (89)	-	
**Use of antibiotics within 12 months**				
No	70.79% (286)	69.75% (113)	71.49% (173)	0.738
Yes	29.21% (118)	30.25% (49)	28.51% (69)	
**Training or workshop on antibiotic use**				
No	88.61% (358)	87.04% (141)	89.67% (217)	0.428
Yes	11.39% (46)	12.96% (21)	10.33% (25)	

HS = Health Science, NHS = Non-Health Science, SD = Standard Deviation

### Sources of information

The academic curriculum was the most prevalent source of information/form of communication about antibiotics among the students from both groups, followed by the internet, medical doctors, pharmacists, television or radio, and friends or family (Table 2). The HS students learned significantly more about antibiotics from the academic curriculum, whereas the NHS students learned more from the internet. The academic curriculum was found to have a significant correlation with the KAP levels among the university students (P < 0.001). There was a statistically significant correlation between communication via the academic curriculum and practices among the HS students (P = 0.027).

**Table 2:** Sources of information about antibiotics use and resistance.

Sources of Information	% (n) Total (N = 404)	% (n) HS (N = 162)	% (n) NHS (N = 242)	*P*
Academic curriculum	55.20% (223)	83.33% (135)	36.36% (88)	< 0.001
Television or Radio	27.23% (110)	38.72% (62)	19.83% (48)	0.425
Internet or social media	53.22% (215)	51.85% (84)	54.13% (131)	0.685
Medical Doctor	39.60% (160)	44.44% (72)	36.36% (88)	0.120
Pharmacist	38.12% (154)	42.59% (69)	35.12% (85)	0.144
Friend or Family	23.27% (94)	20.37% (33)	25.21% (61)	0.281
Other	8.42% (34)	8.02% (13)	8.68% (21)	0.857

HS = Health Science, NHS = Non-Health Science

### Knowledge level

Overall, the average knowledge score among students was 10.73 ± 2.50. HS students had a higher average score of 12.00 ± 2.70, compared to NHS students who scored an average of 9.88 ± 1.94 (P<0.05). Among the most significant findings regarding knowledge (Table 3) was that up to 80% of all the students believed that antibiotics are used to prevent infectious diseases, and more than 75% stated that antibiotics can kill viruses. More than half of both the HS and NHS students believed that antibiotic-resistant bacteria (ARB) cannot be transmitted from person to person or animal to person as a result of antibiotic use to treat humans and animals. Over 65% of the students considered antibiotic resistance to be an individual problem that only affects people who use antibiotics and has no impact on the environment or community.

**Table 3:** Knowledge of antibiotic use and resistance in health science (n = 162) and non-health science (n = 242) students.

Knowledge questions	Area of study	Response, Number (%)		*P* ^a^
		True	False	
Antibiotics are effective in preventing infectious diseases.	HS	134 (82.72%)	28 (17.28%)	0.324
	NHS	209 (86.36%)	33 (13.64%)	
Antibiotics are effective in reducing fever and allergies.	HS	74 (45.68%)	88 (54.32%)	<0.001
	NHS	158 (65.29%)	84 (34.71%)	
Antibiotics are effective in the treatment of viral infections.	HS	113 (69.75%)	49 (30.25%)	0.002
	NHS	201 (83.06%)	41 (16.94%)	
Antibiotics are effective in the treatment of bacterial infections.	HS	149 (91.98%)	13 (8.02%)	0.395
	NHS	216 (89.26%)	26 (10.74%)	
Antibiotics are anti-inflammatory drugs that can alleviate aches and pains caused by hard work, sports, and other activities.	HS	52 (32.10%)	110 (67.90%)	<0.001
	NHS	127 (52.48%)	115 (47.52%)	
Antibiotics can be used without prior medical consultation.	HS	2 (1.23%)	160 (98.77%)	0.212
	NHS	9 (3.72%)	233 (96.28%)	
Antibiotics can be reduced or suspended as soon as symptoms disappear.	HS	48 (29.63%)	114 (70.37%)	<0.001
	NHS	131 (54.13%)	111 (45.87%)	
If you forget to take your antibiotics as scheduled, you take the drug as soon as you remember to, followed by another pill at the next meal.	HS	116 (71.60%)	46 (28.40%)	0.086
	NHS	153 (63.22%)	89 (36.78%)	
Antibiotics can be used for as long as needed without causing any adverse health effects.	HS	26 (16.05%)	136 (83.95%)	<0.001
	NHS	91 (37.60%)	151 (62.40%)	
Antibiotics used in low doses take longer to treat the disease.	HS	53 (32.72%)	109 (67.28%)	0.001
	NHS	119 (49.17%)	123 (50.83%)	
Antibiotics that have been used can be stored until the next time you experience the same symptoms.	HS	40 (24.69%)	122 (75.31%)	<0.001
	NHS	112 (46.28%)	130 (53.72%)	
After taking antibiotics, the drug is excreted through urine or feces.	HS	136 (83.95%)	26 (16.05%)	0.160
	NHS	189 (78.10%)	53 (21.90%)	
Disposing of or burying any leftover or expired antibiotics can cause drug residues in nature	HS	119 (73.46%)	43 (26.54%)	0.652
	NHS	172 (71.07%)	70 (28.93%)	
An incomplete course of antibiotic treatment prescribed by a doctor contributes to antibiotic resistance.	HS	153 (94.44%)	9 (5.56%)	<0.001
	NHS	188 (77.69%)	54 (22.31%)	
Antibiotic-resistant bacteria can spread from person to person.	HS	77 (47.53%)	85 (52.47%)	0.152
	NHS	97 (40.08%)	145 (59.92%)	
Antibiotic-resistant bacteria can spread between animals and humans.	HS	71 (43.83%)	91 (56.17%)	0.412
	NHS	96 (39.67%)	146 (60.33%)	
Antibiotic resistance is only a problem for people who take antibiotics.	HS	105 (64.81%)	57 (35.19%)	0.026
	NHS	182 (75.21%)	60 (24.79%)	
If bacteria are resistant to antibiotics, it can be very difficult to treat the infections they cause.	HS	150 (92.59%)	12 (7.41%)	0.003
	NHS	198 (81.82%)	44 (18.18%)	

^a^ The P-value was calculated using the Fisher extract test.

The HS students demonstrated a significantly greater understanding of several topics than their NHS counterparts (P < 0.05), including those regarding the goals of antibiotic use, the inappropriate use of antibiotics, and the effects of antibiotic resistance. The transformed knowledge scale was significantly higher (P < 0.001) for the HS students (66.67 [22.22–100.00]) than the NHS students (54.87 [22.22–88.89]) (Table 6); the score was higher (P < 0.001) among the fourth year students in both academic programs (63.94 [27.78–100.00]) than those in their first year (59.30 [38.89–83.33]), second year (56.52 [22.22–88.89]), and third year (55.56 [22.22–88.89]). The correlation between knowledge scores and academic year was observed solely among HS students (P<0.05), with no such correlation identified among NHS students. Furthermore, there were no significant differences in terms of the other demographic factors, antibiotic use, and antibiotic training experience.

### Attitude

The average attitude score of the HS students (39.22 ± 4.64) was higher than that of the NHS students (35.91 ± 4.18) (P<0.05), while the average score for all the students was 37.24 ± 4.66. In terms of attitudes, a significant difference was evident between the HS and NHS students (P < 0.05) for almost every topic (Table 4). Approximately 45% of the NHS students thought antibiotics that had previously been effective should be purchased again to reduce costs, and the leftover antibiotics should be kept for future use. A critical issue is that up to 50% of all the students agreed that antibiotics can be used to prevent infection, which is the same as the result revealed in the knowledge assessment. More than 85% of all the students believed that scheduled vaccinations could reduce antibiotic use, and approximately 75% of the respondents stated that infectious illnesses are more severe and cannot be treated with the same antibiotics. Despite the fact that attitudes toward antibiotic resistance and resistance resolution differed significantly between the HS and NHS students, nearly 80% of the NHS students had positive attitudes with respect to these issues. The transformed attitude scale was significantly higher (P < 0.001) for the HS students (73.04 [50.00–100.00]) than the NHS students (64.78 [25.00–100.00]) (Table 6). The correlation between attitude scores and academic year was observed solely among HS students (P<0.05), with no such correlation identified among NHS students. Furthermore, there were no significant differences in the other demographic factors, academic year, antibiotic use, and antibiotic training experience.

**Table 4:** Attitudes of antibiotic use and resistance in health science (n = 162) and non-health science (n = 242) students.

Question (correct answer)	Area of study	Response, Number (%)					*P*
		Largely agree	agree	Uncertain	Disagree	Largely disagree	
Do you agree that vaccinations can help reduce the use of antibiotics?	HS	71 (43.83%)	78 (48.15%)	10 (6.17%)	1 (0.62%)	2 (1.23%)	0.078
	NHS	80 (33.06%)	131 (54.13%)	24 (9.92%)	6 (2.48%)	1 (0.41%)	
Do you agree that antibiotics can be used to prevent infectious diseases?	HS	43 (26.54%)	57 (35.19%)	20 (12.35%)	24 (14.81%)	18 (11.11%)	<0.001
	NHS	54 (22.31%)	132 (54.55%)	40 (16.53%)	14 (5.79%)	2 (0.83%)	
Do you agree that everyone should take the full course of antibiotics as prescribed by the doctor rather than stopping when they feel better?	HS	116 (71.60%)	31 (19.14%)	9 (5.56%)	6 (3.70%)	0 (0.00%)	<0.001
	NHS	95 (39.26%)	111 (45.87%)	26 (10.74%)	7 (2.89%)	3 (1.24%)	
Do you agree that people should buy the same antibiotic that works every time to save money?	HS	20 (12.35%)	28 (17.28%)	37 (22.84%)	45 (27.78%)	32 (19.75%)	0.004
	NHS	33 (13.64%)	80 (33.06%)	51 (21.07%)	51 21.07%)	27 (11.16%)	
Do you agree that buying antibiotics without a prescription will result in a failure to control antibiotic use?	HS	46 (28.40%)	47 (29.01%)	24 (14.81%)	27 (16.67%)	18 (11.11%)	0.014
	NHS	50 (20.66%)	99 (40.91%)	50 (20.66%)	27 (11.16%)	16 (6.61%)	
Do you agree that any leftover antibiotics should be kept for future use to treat other ailments?	HS	15 (9.26%)	20 (12.35%)	20 (12.35%)	44 (27.16%)	63 (38.89%)	<0.001
	NHS	26 (10.74%)	85 (35.12%)	39 (16.12%)	56 (23.14%)	36 (14.88%)	
Do you agree that recent infectious diseases have become more severe and cannot be treated with the same antibiotics?	HS	54 (33.33%)	72 (44.44%)	23 (14.20%)	10 (6.17%)	3 (1.85%)	0.346
	NHS	61 (25.21%)	116 (47.93%)	48 (19.83%)	14 (5.79%)	3 (1.24%)	
Do you agree that the inappropriate use of antibiotics is the main cause of antibiotic resistance?	HS	90 (55.56%)	55 (33.95%)	9 (5.56%)	5 (3.09%)	3 (1.85%)	<0.001
	NHS	67 (27.69%)	119 (49.17%)	41 (16.94%)	11 (4.55%)	4 (1.65%)	
Do you agree that the solution to antibiotic misuse must be implemented in all sectors, both in the public and private sectors?	HS	85 (52.47%)	62 (38.27%)	10 (6.17%)	4 (2.47%)	1 (0.62%)	<0.001
	NHS	72 (29.75%)	124 (51.24%)	39 (16.12%)	7 (2.89%)	0 (0.00%)	
Do you agree that knowledge of antibiotic use and resistance should be included in the curriculum so that all students receive instruction or training?	HS	106 (65.43%)	45 (27.78%)	10 (6.17%)	1 (0.62%)	0 (0.00%)	<0.001
	NHS	91 (37.60%)	109 (45.04%)	38 (15.70%)	3 (1.24%)	1 (0.41%)	

^a^ The P-value was calculated using the Fisher extract test.

### Practice

A small proportion of the students (12.87%, 52/404) reported antibiotic use within the previous 12 months. Among these, the most commonly used antibiotic was amoxicillin (7.43%, 30/404), followed by amoxicillin/clavulanate (3.96%, 16/404), penicillin (2.72%, 11/404), and ciprofloxacin (0.74%, 3/404). Additionally, 45.05% (182/404) of the university students reported obtaining their antibiotics from a doctor in a hospital or clinic, while 46.04% (186/404) mentioned acquiring antibiotics from a pharmacist in a drugstore. Notably, a portion of the students (19.06%, 77/404) acknowledged obtaining antibiotics from inappropriate sources, including friends and family, leftover medications, and online sources.

The students achieved an average practice score of 15.67 ± 3.69. Notably, HS students exhibited a higher average score of 17.24 ± 3.86, whereas NHS students scored an average of 14.62 ± 3.17 (P<0.05). Among the important practice results (Table 5), there was a significant difference in antibiotic use between the HS and NHS students (P < 0.05) with exception of leftover antibiotic waste management. More than 50% of the NHS students had used antibiotics for muscle pain and inflammation, coughing, and headaches. Our findings revealed that up to 65% of all the students had stopped taking antibiotics when they felt better, while 69% had never taken a double dose of medication if they had forgotten to take a dose of the antibiotic. Additionally, approximately 60% of the HS students had not purchased antibiotics unless prescribed by a doctor, whereas 62.81% of the NHS students had purchased the drug on their own based on previous use. In terms of general practice, 60%–70% of the HS students always took antibiotics in accordance with the doctor or pharmacy’s instructions and the drug label, whereas NHS students demonstrated a lower rate, ranging from 42% to 47%.

**Table 5:** Practices of antibiotic use and resistance in health science (n = 162) and non-health science (n = 242) students.

Question (correct answer)	Area of study	Response, Number (%)			*P*
		Always	Sometime	Never	
Have you ever taken antibiotics for muscle or joint pain?	HS	7 (4.32%)	64 (39.51%)	91 (56.17%)	<0.001
	NHS	20 (8.26%)	138 (57.03%)	84 (34.71%)	
Have you ever taken antibiotics for a stuffy nose or a headache?	HS	13 (8.02%)	63 (38.89%)	86 (53.09%)	0.002
	NHS	29 (11.98%)	128 (52.89%)	85 (35.13%)	
If you forget to take an antibiotic, you double the next dose.	HS	7 (4.32%)	20 (12.35%)	135 (83.33%)	<0.001
	NHS	17 (7.02%)	78 (32.23%)	147 (60.75%)	
Have you ever suspended antibiotics use after your symptoms have disappeared?	HS	19 (11.73%)	67 (41.36%)	76 (46.91%)	0.001
	NHS	34 (14.05%)	139 (57.44%)	69 (28.51%)	
Have you ever kept leftover antibiotics with the intention of future use?	HS	11 (6.79%)	45 (27.78%)	106 (65.43%)	<0.001
	NHS	24 (9.92%)	115 (47.52%)	103 (42.56%)	
Have you ever bought the same antibiotics as previously prescribed by a doctor to treat infections?	HS	19 (11.73%)	48 (29.63%)	95 (58.64%)	<0.001
	NHS	23 (9.50%)	129 (53.31%)	90 (37.19%)	
Have you ever bought antibiotics based on old antibiotic packaging or samples of drugs that have been used to treat illnesses?	HS	14 (8.64%)	47 (29.01%)	101 (62.35%)	
	NHS	24 (9.92%)	107 (44.21%)	111 (45.87%)	
Have you ever taken antibiotics as prescribed by a doctor or pharmacist?	HS	115 (70.99%)	33 (20.37%)	14 (8.64%)	<0.001
	NHS	114 (47.11%)	87 (35.95%)	41 (16.94%)	
Before taking antibiotics, you carefully read the label to ensure that it can treat your disease or symptoms and to be sure how to take it.	HS	99 (61.11%)	47 (29.01%)	16 (9.88%)	0.002
	NHS	104 (42.98%)	103 (42.56%)	35 (14.46%)	
Have you ever informed your family and friends about the risks associated with the use of non-prescribed antibiotics?	HS	54 (33.33%)	69 (42.59%)	39 (24.07%)	0.003
	NHS	44 (18.18%)	122 (50.41%)	76 (31.40%)	
When someone is going to self-medicate with antibiotics, I try to persuade them not to do it.	HS	58 (35.80%)	69 (42.59%)	35 (21.61%)	0.002
	NHS	47 (19.42%)	127 (52.48%)	68 (28.10%)	
Have you ever thrown away any leftover antibiotics with other trash?	HS	28 (17.28%)	79 (48.77%)	55 (33.95%)	0.503
	NHS	44 (18.18%)	129 (53.31%)	69 (28.51%)	

^a^p-value was calculated using Fisher extract test.

Only 30% of the HS students had educated their family and friends on the use and effects of antibiotics. The transformed practice scale was significantly higher (P < 0.001) for the HS students (71.84 [100.00–33.33]) than the NHS students (60.90 (95.83–29.17]) (Table 6); the score was higher (P < 0.05) among the fourth-year students of both academic streams (68.64 [100.00–33.33]) than those in their first year (64.74 [100.00–33.33]), second year (63.22 [95.83–33.33]), and third year (62.10 [91.67–29.17]). The correlation between practice scores and academic year was observed solely among HS students (P<0.05), with no such correlation identified among NHS students. There were also no significant differences in the other demographic factors, antibiotic use, and antibiotic training experience.

**Table 6:** Knowledge, attitudes and practices indexes with respect to demographic characteristics (Mann-Whitney and Kruskal Wallis test).

	Knowledge Mean (IQR)	Attitude Mean (IQR)	Practice Mean (IQR)
**Academic program** *	<0.05	<0.05	<0.05
Health Science	66.67 (22.22–100.00)	73.04 (50.00–100.00)	71.84 (33.33–100.00)
Non-Health Science	54.87 (22.22–88.89)	64.78 (25.00–100.00)	60.90 (29.17–95.83)
**Sex** *	0.813	0.720	0.224
Male	59.62 (22.22–100.00)	67.98 (25.00–100.00)	64.22 (29.17–100.00)
Female	59.59 (27.78–100.00)	68.17 (40.00–100.00)	56.98 (33.33–100.00)
**Religion** **	0.811	0.481	0.913
Buddhism	59.46 (22.22–100.00)	68.23 (25.00–100.00)	65.35 (29.17–100.00)
Islam	59.81 (22.22–100.00)	67.19 (50.00–92.50)	64.81 (33.33–100.00)
Other	63.89 (50.00–77.78)	73.33 (52.50–100.00)	68.06 (37.50–91.67)
**Academic Year** **	<0.05	0.75	<0.05
1	59.30 (38.89–83.33)	66.25 (42.50–85.00)	64.74 (33.33–100.00)
2	56.52 (22.22–88.89)	68.40 (47.50–90.00)	63.22 (33.33–95.83)
3	55.56 (22.22–88.89)	65.79 (25.00–92.50)	62.10 (29.17–91.67)
4	63.94 (27.78–100.00)	70.15 (40.00–100.00)	68.64 (33.33–100.00)
**Use of antibiotic within 12 months** *	0.795	0.844	0.071
No	59.52 (22.22–100.00)	68.14 (25.00–100.00)	66.26 (33.33–100.00)
Yes	59.79 (22.22–94.44)	67.99 (40.00–90.00)	62.92 (29.17–100.00)
**Training or workshop on antibiotic use** *	0.599	0.096	0.515
Never	59.37 (22.22–100.00)	67.63 (25.00–100.00)	65.27 (29.17–100.00)
Ever	61.72 (33.33–94.44)	71.68 (50.00–100.00)	65.40 (33.33–100.00)

* Mann-Whitney U test

** Kruskal-Wallis test

### The correlation of KAP in university students

The correlations between the antibiotic KAP among the university students in southern Thailand are shown in Table 7. Significant positive correlations were found between knowledge and attitude (rho = 0.398), knowledge and practice (rho = 0.506), attitude and practice (rho = 0.429). When comparing the results of KAP among HS students, we observed a positive correlation between knowledge and attitude (rho = 0.434), knowledge and practice (rho = 0.477), and attitude and practice (rho = 0.447). Additionally, among NHS students, we also found positive correlations between knowledge and attitude (rho = 0.191), knowledge and practice (rho = 0.376), and attitude and practice (rho = 0.244).

**Table 7:** Spearman’s correlation between KAP-level.

	**Knowledge**	**Attitude**	**Practice**
**Knowledge**	1		
**Attitude**	0.398**	1	
**Practice**	0.506**	0.429**	1

**Correlation is significant at the 0.01 level (2-tailed).

*Correlation is significant at the 0.05 level (2-tailed).

## Discussion

In the present study, we showed the results of the KAP in relation to antibiotic use and resistance among university students in Thailand. The majority of the students had become ill with the flu (48.27%) in the previous 12 months, followed by diarrhea and tonsillitis. Only 12.87% of the students took antibiotics. Amoxicillin was the most commonly used antibiotic among the students, followed by amoxicillin/clavulanate, penicillin, and ciprofloxacin. Most of the students (45.05%) obtained antibiotics from a doctor in a hospital or clinic and 46.04% from a pharmacist in a drugstore, while some of the students (19.06%) obtained antibiotics from friends, family, residual drugs, and the internet. Furthermore, the students learned about antibiotic usage and resistance in the classroom (55.20%), online and on social media (53.20%), and from doctors (39.60%) and pharmacists (38.10%). These findings are consistent with those of previous studies based on the national household surveys on antibiotic knowledge and use in Thailand [14, 18] in which Thai adults reported high levels of flu and a low prevalence of antibiotic use (7.9%). Over 90% of the respondents received antibiotics from healthcare professionals, and doctors (36.1%), health workers (42.50%), and pharmacists (17.7%) were the most common sources of information about rational antibiotic use and antibiotic resistance.

In terms of KAP regarding antibiotic use and resistance in our study, the HS students scored significantly higher than the NHS students (P < 0.001). Previous comparative studies have similarly revealed a significant difference in KAP scores for antibiotic use and resistance between medical students (MSs) and non-medical students (NMSs) [17, 32, 33], biological science and non-biological science students [30] and students with health-related majors and non-health-related majors [22, 34]. The results of the present study, along with those reported in previous studies, indicate that study programs have a significant influence on KAP levels. Students enrolled in HS programs are the future health professionals who will play a critical role in controlling antibiotic use and preventing antibiotic resistance. However, antibiotic use and resistance KAP should therefore be promoted to HS and NHS students.

Among the students in our study, the academic curriculum was the main source of information on antibiotic use and resistance, and this finding was significantly higher among the HS students, which is similar to the results of a comparative study among veterinary students and non-veterinary students in Bangladesh [27]. In the context of evaluating the curriculum’s impact through a comparison between HS and NHS students, it is evident that HS students demonstrated superior knowledge levels concerning antibiotic use and resistance. A comparative study in Bangladesh determined that biological science students who learned about antibiotics were more knowledgeable than non-biological science students [30]. Furthermore, the students who received knowledge in the classroom demonstrated more positive attitudes and practices than those who did not. Similar findings in Colombia revealed that MSs with prior experience in antibiotic research or education had higher KAP levels with regard to antibiotic use and resistance than NMSs [40].

In our study, the students’ academic year was found to be correlated with their KAP regarding antibiotic use and resistance. The senior students would have received more knowledge through classes, workshops, and training than freshman. Additionally, our findings revealed a significant correlation between academic year and KAP scores in HS students. Similar findings were found in China in 2013, which revealed improved knowledge and attitudes toward antibiotic use as MSs’ grades increased [33].

The results of our study provide valuable insights into the relationships between knowledge, attitudes, and practices (KAP) regarding antibiotic use and resistance among both Health Science (HS) and Non-Health Science (NHS) students. A correlation between KAP in relation to AMR has been reported among Chinese public health students [25] and those in Bangladesh [27]. In another study, an evaluation of the KAP regarding antibiotic use among community members revealed a positive correlation between knowledge and practices, as well as a positive correlation between attitudes and practices [13]. Previous studies on the correlation between KAP have suggested that increasing students’ knowledge of antibiotic use may help improve and rationalize their attitudes toward AMR [7, 34]. In contrast, the study conducted among Maasai pastoralists in Tanzania did not find a correlation between knowledge and attitude, and practice [41].

The knowledge survey findings in our study revealed that only 7.67% of the HS and 0.99% of the NHS students had a strong understanding of antibiotic use and resistance. Based on their responses to the questions, we demonstrated that the students misunderstood several of the topics. Over 80% of the HS and NHS students thought that antibiotics could be utilized for disease prevention, and this perception did not exhibit a significant difference between the two groups. The majority of the students believed that antibiotics are effective in treating viral infections (77.72%) and fever (57.43%). These results are similar to those of the national household survey on antibiotic knowledge and use in Thailand as a large proportion of those respondents did not know that antibiotics do not kill viruses (80.60%) or cure the common cold (79.80%) [14]. More than half of the Thai population thought antibiotics were anti-inflammatory drugs that can be used to relieve pain, which is consistent with the results of this study. A high percentage (84.40%) of the students in our study understood that improper antibiotic use leads to resistance, which is consistent with the findings of the Thai population survey (63.60%) [14]. A study in Lebanon found a similar percentage of HS and NHS students (43.30% and 37.20%, respectively) correctly responded to a question about the effectiveness of antibiotics to treat fever [34]. In comparison to our findings, the results from the Lebanon study revealed a high number of HS (75.20%) and NHS (81.80%) students who could correctly answer the question regarding antibiotic resistance transmission. Moreover, the HS and NHS students knew when to stop using antibiotics (95% and 80.7%, respectively), which was a higher percentage than in this study (70.40% and 45.90%, respectively). Compared to 82% of the undergraduate students in a study in the United States, a lower proportion of the students in this study (43.07%) knew that ARB can be spread between people [28]. Our findings, along with those of several other studies, revealed a significant difference in knowledge about the proper use of antibiotics between MSs and NMSs [17, 32, 33] as well as between HS and NHS students.

More HS students in this study (48.77%) had positive and satisfactory attitudes compared to those of the NHS students (17.36%). The HS students provided more correct answers than the NHS students with regard to rational antibiotic use, resistance to irrational antibiotic use, antibiotic resistance solutions, and antibiotic knowledge increases through classroom instruction. On the other hand, the NHS students had strong negative attitudes toward the acquisition of antibiotics. In terms of attitudes toward antibiotic use and resistance, 84.90% of all the students agreed that antibiotic misuse and resistance were complex issues that need to be addressed by multiple sectors. In comparison, only 28.50% of the students in a study in Colombia recognized these issues as a multifactorial problem that could not be solved by individuals [40]. In both studies, a small number of the students thought that they could stop taking antibiotics once their symptoms went away. Notwithstanding, our findings revealed a higher percentage of students (81.93%; HS students = 89.60%, NHS students = 76.90%) with positive attitudes toward antibiotic resistance than a study in Lebanon, which found the same to be true for 77.73% of the students (89.10% of the HS students and 57.88% of the NHS students) [34]. Furthermore, a high number of the Lebanese university students correctly answered the question about buying the same antibiotics to help treat the same symptoms they had experienced in the past (84% vs. 68.2% for health- and non-health-related majors) [34], while in our study, a low rate of students showed a positive attitude on this issue (19.05% vs. 19.30% for the HS and NHS students, respectively).

Among the HS students in our study, 41.36% indicated that they followed good practices regarding antibiotic use and resistance compared to the NHS students (10.74%). Almost all the antibiotics practices among the HS students were significantly more appropriate than those of the NHS students, with exception of the waste management of leftover antibiotics. However, several instances of improper antibiotic use were discovered in this group of participants. Indeed, 48.27% (195/404) of all the students kept leftover antibiotics for future use although this was mostly the case among the NHS students at 71.28% (139/195). The majority of the students (64.11%, 259/404) had stopped taking antibiotics when their symptoms had disappeared, with the percentage of NHS students doing so at 66.80% (173/259). In terms of waste management, 69.31% of the students (HS students = 66.05%, NHS students = 71.50%) had thrown the leftover antibiotics in the garbage along with other trash. Antibiotic misuse among university students has also been reported in several other parts of the world. In a study in Nigeria, more than half of the students had kept leftover antibiotics for future use, and 55% had not finished their antibiotic courses [7]. The majority of veterinary and non-veterinary students in Bangladesh managed leftover antibiotics by throwing them away (40.76% vs. 42.61%, respectively), followed by keeping them for future use (19.33% vs. 16.92%, respectively) [27]. Studies involving students in Bangladesh (41.95%), the United States (38.00%), and Nepal (85.70%) reported that the students stopped taking antibiotics once their symptoms had disappeared [28, 30, 32].

The exploration of antibiotic use and resistance extends beyond factual knowledge, encompassing individuals’ attitudes and beliefs, which are often intertwined. It is imperative to acknowledge that the overlaps among specific questions within the Knowledge, Attitudes, and Practices (KAP) framework can be attributed to the intricate and multifaceted nature of antibiotic-related knowledge and attitudes. Antibiotic utilization and resistance encompass a spectrum of facets, including medical insights, societal conventions, and individual conduct. Consequently, certain questions may exhibit surface-level similarities, they are designed to probe distinct dimensions of participants’ comprehension and behavior.

In our study, the students reported rational antibiotic use, including always taking antibiotics as prescribed by a doctor (71.00% of the HS students vs. 47.10% of the NHS students). Previous studies in Bangladesh and Nepal found that up to 90% of students had taken antibiotics as prescribed by their doctor [30, 32], while 66.40% of the students in Yemen had used antibiotics without a prescription [31].

Antibiotic resistance is a multifaceted problem that requires collaboration between all sectors to ensure information is shared and effective policies developed. Thailand developed the National Strategic Plan on Antimicrobial Resistance 2017–2021, which is the first country’s national strategy aimed specifically at addressing AMR issues. One important goal of the national strategic plan is to increase public knowledge about AMR and awareness of rational antimicrobial use. The outcomes of improved knowledge and awareness could promote rational antimicrobial use and reduce antimicrobial consumption. The dissemination of adequate knowledge through education is an important intervention that is necessary to achieve the national goal of antimicrobial stewardship. As suggested by medical and pharmacy students, comprehensive training on antibiotic use could help them when prescribing and dispensing appropriate antibiotics [19].

Educating university students on antibiotic use and resistance is one of the strategies for antimicrobial stewardship in both hospitals and communities. Several studies conducted around the world have recommended that university students receive more education about antibiotic use and resistance. For example, university students in southwestern Nigeria need better education on the rational use of antibiotics to improve their attitudes toward antibiotics [7]. In India, there is a need for more educational programs for students and interns not only to enhance their knowledge, but also to reshape the awareness and behavior of the students regarding antibiotic use [26]. Studies in Nepal and the United Arab Emirates have suggested that it is necessary to establish specific courses on rational antibiotic use in academic curricula [17, 32]. In Bangladesh, introducing a short course on the risks and development of antibiotic resistance has been suggested to increase students’ awareness of how to avoid the resistance phenomenon [30]. In the United States, health education about antimicrobial stewardship has been recommended to increase knowledge, perceptions, health behaviors, and social norms on antibiotic use [28], while a study in Yemen revealed the need to increase students’ awareness of proper antibiotic use through adequate educational programs [31]. In Columbia, there is a need to strengthen the curriculum of MSs on antibiotics, the mechanisms of antibiotic resistance, and the prudent use of antibiotics as an important strategy to combat problem-resistant public health issues [40]. Furthermore, antibiotic resistance and antibiotic stewardship courses, seminars, and workshops should be introduced into the curricula of all programs to improve students’ practices on antibiotic use, as suggested by previous studies in East Africa [19], China [25, 33], the United Arab Emirates [17], and Lebanon [34].

Based on the findings of our study, academic curricula in Thailand can influence the KAP regarding antibiotic use and resistance among university students, as has been reported in China [33], Bangladesh [30], Colombia [15] and Yemen [31]. The correlation between classroom education and KAP levels in this study may highlight the importance of course curricula as a critical source for delivering knowledge about antibiotic use and resistance to both HS and NHS students. Such knowledge dissemination may encourage rational antibiotic use and prescribing by students, which comprise one of the most important populations that can help prevent and reduce the burden of antibiotic resistance.

This study had some limitations, most notably related to the sample groups, sample size, and data collection method. We surveyed the KAP regarding antibiotic use and resistance among HS and NHS students across all schools at WU, with the exception of the students in the dental and veterinary colleges. Because these two schools were only established two years ago, unlike the other schools, they had no third- and fourth-year students. The HS students’ KAP results may therefore not reflect the most recent information available from this university.

Although the data were collected as planned, larger sample sizes and the inclusion of more university and non-university students will be required in future studies to better understand the KAP levels regarding antibiotic use and resistance among teenagers and young adults in Thailand. Furthermore, we acknowledge the potential for bias in our study. Specifically, with regards to response bias, participants in the electronic self-administered survey were volunteers, which may have attracted students who are more knowledgeable or interested in the topic. This could lead to an overrepresentation of individuals with better knowledge and practices regarding antibiotic use and resistance. Additionally, there may be a selection bias due to the voluntary nature of participation, where those who were more concerned or informed about the subject may have been more likely to take part, thus potentially skewing the results. Despite its limitations, the findings of this study can serve as a baseline or reference for future studies on this topic and to monitor the effectiveness of future interventions.

## Conclusion

The HS students in our study had significantly better KAP in terms of antibiotic use and resistance than the NHS students. We found that the students’ knowledge scores were positively correlated with their attitudes and practices, and their practices were also positively correlated with their attitudes. KAP scores regarding antibiotic use and resistance increased from the first to the last academic year in both HS and NHS student groups. This finding emphasizes the importance of academic curriculum as a source of antibiotic knowledge for both HS and NHS students. Antibiotic use and resistance knowledge and attitudes should be conveyed to all university students. Such education could set the standard for rational antibiotic use as well as the long-term prevention and control of ARB.
